# Capillary Leak Syndrome Associated with Anaplastic T Cell Lymphoma and Transcutaneous Exudation: An Unusual Presentation

**DOI:** 10.3390/diagnostics14171924

**Published:** 2024-08-31

**Authors:** Radu Andrei Tomai, Antonia Oancea, Ciprian Tomuleasa, Delia Dima

**Affiliations:** 1 Department of Haematology, “Ion Chiricuta” Institute of Oncology, 34–36 Republicii Street, 400015 Cluj-Napoca, Romania; raduandreitomai@gmail.com (R.A.T.); oanceaantonia93@gmail.com (A.O.); deli_dima@yahoo.com (D.D.); 2 Medfuture Research Center for Advanced Medicine, Iuliu Hatieganu University of Medicine and Pharmacy, 400124 Cluj-Napoca, Romania

**Keywords:** capillary leak syndrome, T-cell lymphoma, unusual presentation

## Abstract

Capillary leak syndrome is a rare complication of cancer, particularly of hematologic malignancies. The syndrome was first described as an idiopathic entity; however, increasingly, more cases are being reported in association with cancers and other conditions. Diagnosis stems from the recognition of the double paradox, consisting of severe generalized oedema and hypotension, accompanied by hallmark laboratory modifications. Concurrent conditions in patients with malignancies can alter laboratory findings and make the diagnosis a challenge. This report presents the case of a patient with capillary leak syndrome and an atypical presentation, with generalized skin rash and transcutaneous exudation occurring concurrently with anaplastic large T cell lymphoma, macrophage activation syndrome, and cytopenias. Symptom-specific treatment with diuretics and albumin was ineffective in the case of our patient; however, the CLS remitted promptly with cancer-specific therapy. No treatment has proved to be generally effective against CLS up to date, as is the case for this patient. Thus, the rapid recognition of cancer-associated capillary leak syndrome and the initiation of cancer-specific treatment proves to be the better approach and is key to avoiding unnecessary delays and ineffective treatments targeted specifically at CLS.

A 69 y.o. female patient, with a history of cholecystectomy, presented to the emergency room with extreme fatigue in a post-surgical setting. Two weeks prior, the patient had undergone abdominal surgery for intestinal occlusion due to preexisting fibrosis. The patient was hospitalized in an ICU setting for sepsis of supposed digestive tract origin, where she was treated empirically with Levofloxacin and Metronidazole. A contrast-enhanced thorax–abdomen–pelvis CT scan was performed for the identification of sepsis origin, which revealed multiple retroperitoneal adenopathies up to 2.3/2.6 cm, right iliac adenopathies up to 3.9/2.6 cm, and bilateral inguinal adenopathic blocks up to 3/3.5 cm with central necrosis, as well as a focal hypodense hepatic lesion of 1.3 cm in the 2nd hepatic segment.

An inguinal lymph node biopsy was performed due to suspicion of a lymphoproliferative neoplasm and the patient was transferred to the hematology department for further investigations.

Upon presentation, the patient had an ECOG performance score of 3/5, with extreme fatigue and intermittent fever up to 38.3 °C. Her medical history revealed a recent weight loss of 5 kg within 3 months and the insidious appearance of generalized skin lesions with associated transcutaneous exudation in the past two weeks.

The clinical examination showed grade II obesity, marked pallor, and multiple supraclavicular and axillary adenopathies measuring up to 3 cm in diameter. Remarkably, the patient showed generalized dry, scaly skin lesions with erythematous striae. These were more marked on the lower limbs, with no associated inflammatory signs, and were accompanied by a generalized exudate (Figure 1).

The laboratory results showed leukocytosis of 14,200 × 10^9^/L [4000–10,000 × 10^9^/L], neutrophilia of 13,000 × 10^9^/L [1800–7500 × 10^9^/L], lymphopenia of 740 × 10^9^/L [800–4000 × 10^9^/L], moderate hyporegenerative anemia with hemoglobin of 93 g/L [120–160 g/L], grade II thrombopenia of 73,000 × 10^9^/L [150,000–450,000 × 10^9^/L], inflammatory syndrome with C-reactive protein of 17.6 mg/dL [<0.5 mg/dL], elevated procalcitonin of 2.7 µg/L [<0.5 µg/L], elevated ferritin of 1820 µg/L [13–150 µg/L], hyperfibrinogenemia of 5.6 g/L [1.9–4.3 g/L], elevated LDH of 340 IU/L [<214 IU/L], hypoalbuminemia of 20.5 g/L [35–52 g/L], hypophosphatemia of 0.54 mmol/L [0.80–1.45 mmol/L], hypomagnesemia of 0.51 mmol/L [0.65–1.0 mmol/L], and normal serum protein electrophoresis. Cardiac markers were abnormal, with elevated NT-proBNP 303.9 pmol/L [<55.5 pmol/L]. The coagulation was altered, with a prolonged prothrombin time of 18.3 s [11.4–15.3 s], an APTT of 46 s [24–40 s], and d-dimers of 2200 µg/L [<500 µg/L].

Empirical antibiotic treatment was escalated for large-spectrum coverage, using Imipenem/Cilastatin and Linezolide, while awaiting biopsy results. Urine cultures came back positive for Enterococcus Fecalis with appropriate coverage under the current antibiotic regimen. The inflammatory syndrome remitted under treatment, with the sterilization of urine cultures; however, there was a worsening of cytopenias with a platelet nadir of 22,000 × 10^9^/L and the worsening of ferritin with a peak value of 29,300 µg/L.

The hypothesis of HIT was considered in the context of using low-molecular-weight heparin for post-surgical thromboprophylaxis, but was eventually excluded based on a low probability score. The main additional differential diagnosis was infection or paraneoplastic hemophagocytic lymphohistiocytosis (HLH) and the diagnosis was supported by hyperferritinemia, elevated triglycerides of 6.44 mmol/L [0.4–1.7 mmol/L], and elevated LDH. A bone marrow aspirate was performed to confirm the hypothesis, which showed macrophages laden with cell debris and evident hemophagocytosis. Treatment with a Dexamethasone equivalent of 1 mg/kg Prednisone was started; however, cytopenias persisted and there was no improvement in hemophagocytic syndrome.

Over the next days, there was rapid clinical deterioration associated with the worsening of the skin lesions. The clinical picture was dramatic, with marked diffuse exudation, the development of generalized oedema and anasarca, hypoalbuminemia, and marked hypotension and oliguria. Heart failure decompensation was ruled out and the patient received intravenous fluid resuscitation along with albumin supplementation and diuretics. There was marginal improvement, with a mean arterial blood pressure (MAP) bordering on 65 mmHg; however, 3rd-space fluid sequestration continued to aggravate tissue and was largely refractory to treatment. Examining the patient’s detailed history of medication revealed no recent drug exposure prior to the appearance of skin lesions, and exudate cultures were negative.

Rapid kidney function declined, with a sharp increase in creatinine and oliguria levels following soon after, and the patient developed dyspnea, leukocytosis, and severe thrombopenia, with a nadir of 9000 × 10^9^/L, requiring oxygen supplementation and platelet concentrate transfusions.

In the absence of apparent sepsis and the improvement of inflammatory markers, paralleled by clinical degradation, progressive oedema, hypotension, hypoalbuminemia, and progressive renal dysfunction, with no alternative diagnoses, were suggestive of systemic capillary leak syndrome. The hemoconcentration was difficult to assess, as the patient presented with leukocytosis as well as anemia and thrombopenia; however, in the context of hematologic malignancy, this element was not considered essential for diagnosis as it is often absent in cases with hematologic involvement. Thus, a diagnosis of systemic capillary leak syndrome was made, and the rapid treatment of underlying neoplasia was considered necessary without awaiting the clinical improvement of oedema and kidney function.

The pathology report on the lymph node biopsy was diagnostic of ALK-negative anaplastic large T cell lymphoma, showing the infiltration of medium and large lymphocytes, multinuclear anaplastic cells, hallmark cells with horseshoe-shaped nuclei, and doughnut cells. By immunohistochemical staining, tumor cells were established as CD45+, CD4+, CD30+, perforin+, EMA+, CD5−, CD8−, CD10−, granzyme−, CD20−, CD23−, and ALK−, and found to have high proliferation index values, with a Ki-67 of 80%.

Treatment with Cyclophosphamide, Etoposide, Vincristine, and Prednisone (CEOP) was opted for and started, with doses adapted to hypoalbuminemia and the omission of anthracyclines due to the frailty of the patient. Treatment was well tolerated, with no immediate complications.

Following chemotherapy, there was a rapid improvement in skin lesions, with reductions in and eventually the complete remission of exudate (Figure 2), the dramatic improvement of diuresis with an initial phase of polyuria, the correction of hypoalbuminemia, and the normalization of hemodynamic parameters.

Unfortunately, the patient became less compliant and impatient with hospitalization and requested to be transferred to a different hospital with no dedicated hematology or oncology unit. She was discharged while still in aplasia and did not show up for further follow-up. This is regarded as highly unadvisable, as capillary leak syndrome in patients with hematologic malignancies is associated with increased mortality. The management of such patients in dedicated departments, with knowledge and experience of treatment of hematologic malignancies and their complications, is extremely important as immediate and appropriate care are essential to ensuring the best possible outcomes. Unfortunately, the lack of a follow-up limits the possibility of evaluating the long-term outcomes for this patient and whether capillary leak syndrome as a presentation of T cell lymphoma could have been isolated or whether it would have recurred later.

Capillary leak syndrome is a rare entity, which was first been described by Clarkson as a recurrent idiopathic cause of redistribution hypovolemic shock [1]. It is characterized by endothelial dysfunction and increased vascular permeability. The clinical picture is the result of the loss of protein-rich fluids from vascular spaces to interstitial spaces, leading to a cascade of generalized oedema, hypovolemia, hemoconcentration, hypoalbuminemia, and, in severe cases, hypovolemic shock and multiple organ dysfunction [2]. Three clinical phases have been described for CLS, starting with a prodrome of flu-like symptoms, followed by the characteristic leak phase, and finishing with the resolution phase involving polyuria and resolution of symptoms [3].

There are no clearly defined criteria for the diagnosis for CLS and it ultimately relies on a diagnostic of exclusion for anasarca and distributive shock [4]. Capillary leak syndrome is often missed due to its rarity and the lack of specificity of its symptoms; however, CLS should be taken into consideration upon the recognition of the double paradox of generalized oedema and hypotension 3. The proposed diagnostic criteria are hypotension, hypoalbuminemia, hemoconcentration, and generalized edema [2]. Diagnosis can further be supported by rhabdomyolysis, increased VEGF and angiopoietin-2 levels, and monoclonal gammopathy [5].

In the half century since the syndrome was first described, several hundred cases have been reported including secondary forms of CLS, which are associated with malignancy, anti-neoplastic medication, autoimmunity, hemophagocytosis, gammopathy, and infection [3,6].

Hematologic malignancies are the most common oncologic expressions of CLS. They have been reported in various hematologic malignancies, such as multiple myeloma, anaplastic T cell lymphoma, diffuse large B cell lymphoma, and Waldenstrom disease, and they are associated with increased mortality compared to CLS occurring in other settings [7]. There is a high prevalence of monoclonal gammopathy in these patients; however, immunoglobulins do not appear to play a role in the pathogenesis of this condition [8]. Furthermore, the rate of progression to multiple myeloma is similar to that seen for patients with monoclonal gammopathy of unknown significance [9].

The case described highlights the challenges of the diagnosis and treatment of a patient with CLS associated with anaplastic large T cell lymphoma. The clinical picture in this case was consistent with a diagnosis of CLS with marked hypoalbuminemia, generalized oedema, and hypotension; however, the hemoconcentration was difficult to assess due to the cytopenias.

Numerous confounding comorbidities in patients with cancer can obscure CLS diagnostic features, which leads to the diagnosis often being overlooked. In patients with hematological malignancies, the relevance of hemoconcentration, one of the central laboratory modifications in CLS, is doubtful, as cytopenias develop due to various reasons such as bone marrow infiltration, bleeding, and infections, thus masking the hemoconcentration. In a systematic review of 62 patients with malignancy-related CLS, 48% and 73% of patients presented with anemia and thrombocytopenia, respectively [7].

There is still an unmet need for specific markers of CLS, especially in patients with complex comorbidities, where diagnosis is difficult. Several soluble mediators of CLS have been proposed after the observation of increased levels of CD25+ T cells and CD8+ T lymphocytes; increased serum cytokine levels of CXCL10, CCL2, IL1B, IL-5, IL-8, IL-12, and TNF; and elevated plasma levels of VEGF and angiopoietin. However, none can be consistently used to support the diagnosis [7].

Multiple drugs employed in the treatment of hematologic malignancies, including chemotherapeutics, monoclonal antibodies, and small molecules, may induce CLS. In a systematic review comprising 62 patients with cancer, 51.6% of cases were related to treatment, and 43.6% to the cancer itself 7. While the pathogenesis underlying secondary CLS remains mediated by soluble factors, the cytokines involved seem to differ from those involved in primary CLS. In these settings, CLS is often assimilated with cytokine release syndrome, as a consequence of cytokine storm, and is associated with increased levels of IL-2, TNF-a, IL-4, and IL-6 [2,10].

The overlap of CLS with CRS is even more evident in the case of CAR-T cell therapy. A review of 40 patients that had undergone CAR-T cell therapy revealed the occurrence of CLS in 11 patients, associated with grade 3–4 CRS. In these patients, IL-6 and IFN-gamma levels were significantly higher, and there were markedly increased peak ferritin levels. In such contexts, IL-6 receptor antagonist Tocilizumab may be a useful tool for the treatment of secondary CLS [11].

To date, there is no standard treatment for CLS; however, the mainstay of treatment in CLS is supportive and relies on fluid resuscitation, vasopressors, methylxantines, and diuretics. The choice of treatment is adapted to the clinical presentation, which may be milder, with fluid retention but hemodynamic stability, requiring only diuretics, or severe, with hypotension, shock, and renal impairment [2,7,12].

In patients with hemodynamic instability, the first choice for fluid resuscitation is crystalloid solutions; however, due to the loss of intravascular proteins, some patients have little or no response to crystalloids, which aggravates excess fluid accumulation. One serious complication of severe fluid excess, which may occur, is compartment syndrome. Therapeutic options for these patients who do not respond to crystalloids include albumin and pentastarch, although albumin loss from the intravascular space limits its efficacy. In some cases of secondary CLS, steroids have been successfully used to aid in controlling the disease, which may be related to its occurrence in the setting of CRS [2].

Other treatment approaches aim to counter the presumed pathogenetic mechanisms. The use of the VEGF inhibitor Bevacizumab or the use of intravenous immunoglobulins is based on observations of increased vascular permeability, co-occurrence with increased VEGF, TNF-a levels, and the high proportion of patients with monoclonal gammopathy. Several reports show the benefits of Bevacizumab use, while others show no improvement. Intravenous immunoglobulins are often used to prevent disease recurrence; however, a multicenter retrospective analysis reviewed 59 CLS episodes in 37 patients and failed to show a survival benefit for IVIG use in this setting [12,13].

A particularity of this case, contributing to the severity of the clinical picture, is the presence of transcutaneous exudation. Several different types of cutaneous involvement, such as livedo, sclerosis, purpura, and maculopapular rash, have been described as complications of program death 1 (PD-1) antibodies and in patients with coexisting psoriasis; however, to our knowledge, there are no described cases of capillary leak syndrome with transcutaneous exudation [14]. The loss of proteins through transcutaneous exudation can be an aggravating factor for patients already presenting with hypoalbuminemia.

The urgent need for proper treatment stems from the risk posed by hypovolemia, which can lead to cardiac arrest [3]. Additionally, the post-leak phase poses an important risk due to rapid increases in intravascular volume and fluid overload, which can lead to pulmonary oedema.

It is a common approach in patients with cancers to treat severe complications prior to starting chemotherapy to avoid adding cytotoxicity and cytopenia to an already severe clinical scenario; however, in the case of our patient; the only effective approach to CLS management was treatment of the malignancy. Thus, the recognition of cancer-associated CLS is important for rapid initiation of malignancy-specific therapy as CSL-specific treatment is largely ineffective in these cases and delays appropriate treatment.

The main limitations of this case are the multiple concurrent complications of lymphoma, raising questions regarding which elements of the clinical and laboratory features can be attributed solely to CLS. Additionally, the lack of follow-up makes it impossible to evaluate whether the recurring trait idiopathic CLS is present in cancer-associated CLS as well.

## Figures and Tables

**Figure 1:**
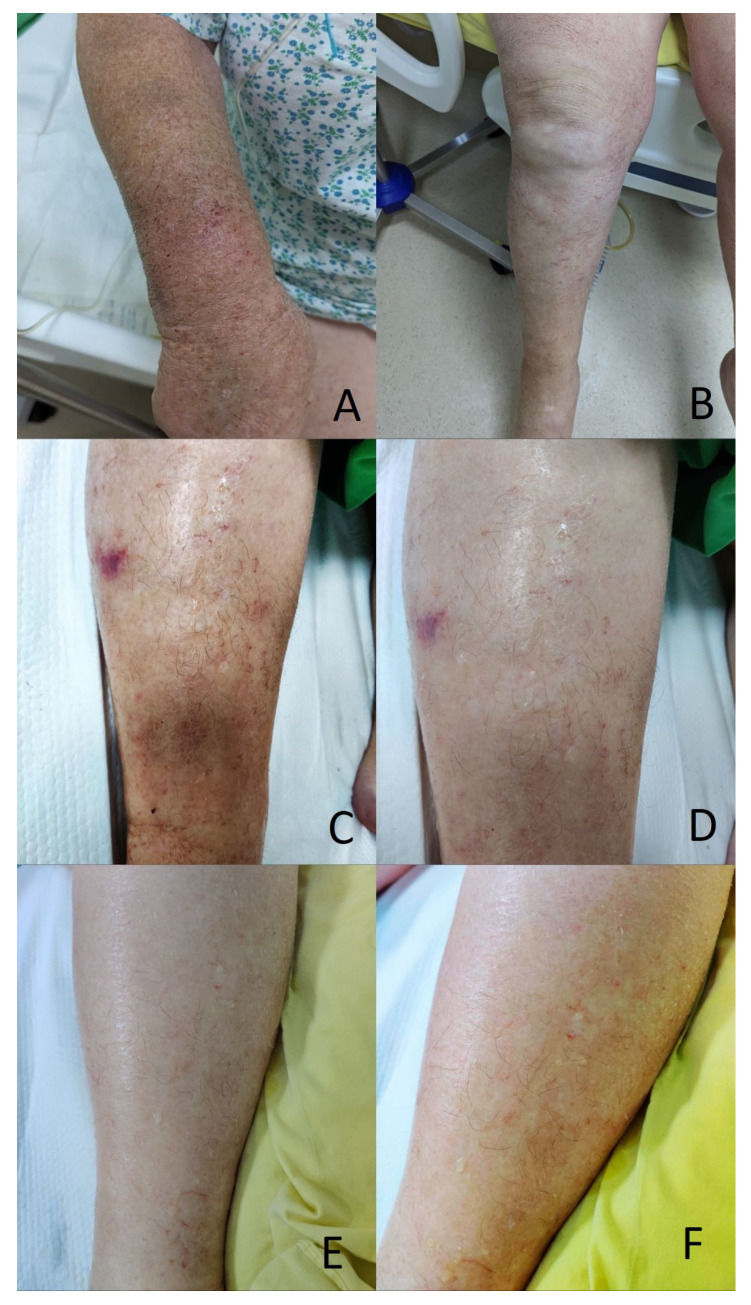
Initial aspects of skin change in the context of capillary leak syndrome and anaplastic T cell lymphoma. (**A**,**B**) scaly erythematous desquamative skin rash with fissures on upper and lower limbs. (**C**–**F**) close-up of lower limbs, showing marked symmetric oedema and diffuse transcutaneous exudation irrespective of skin lesions.

**Figure 2:**
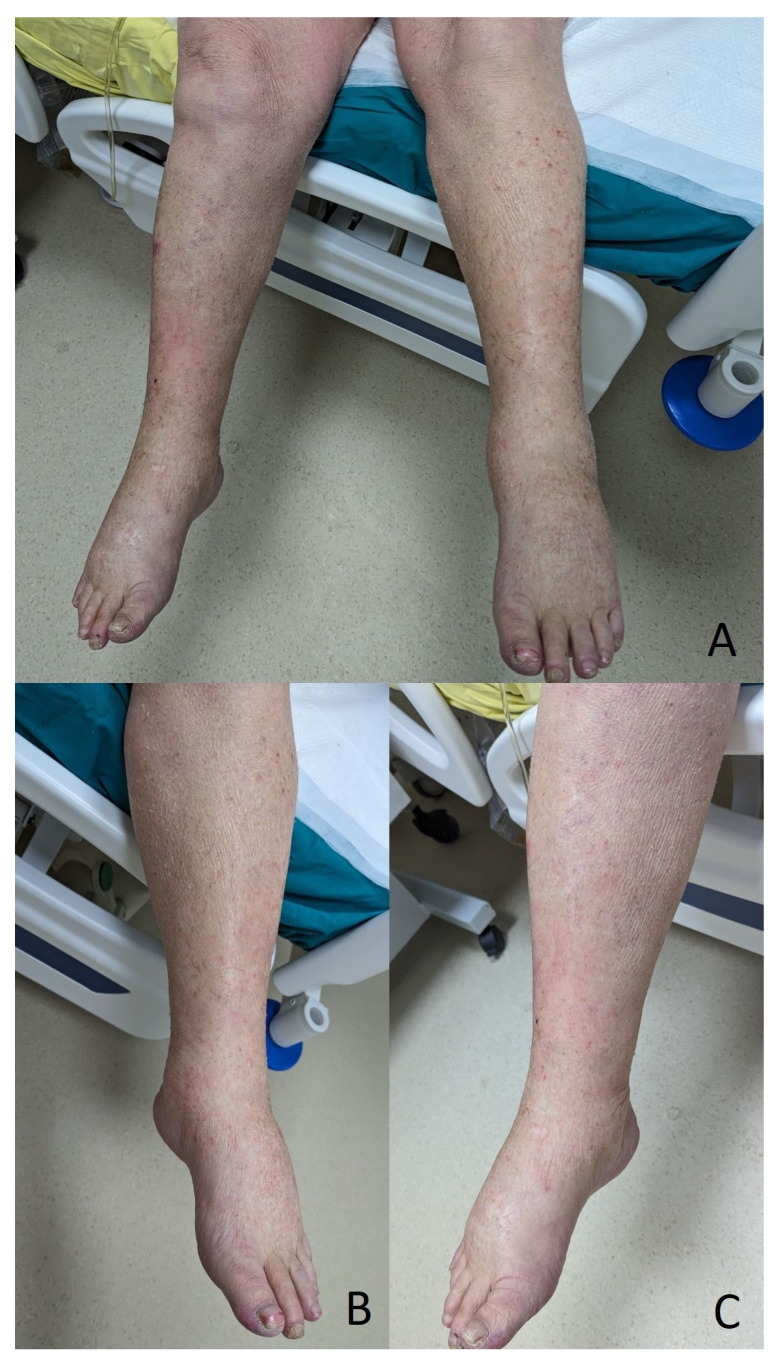
Aspects of skin change following chemotherapy and improvement of capillary leak syndrome. (**A**) Improvement of skin fissures and persistence of dry scaly aspects following remission of generalized oedema. (**B**,**C**) Close-up of lower limbs, showing improvement in skin changes and rashes with remission of transcutaneous exudation.

## Data Availability

All data is available upon reasonable request.

## References

[1] Clarkson B., Thompson D., Horwith M., Luckey E.H. (1960). Cyclical edema and shock due to increased capillary permeability. Am. J. Med..

[2] Siddall E., Khatri M., Radhakrishnan J. (2017). Capillary leak syndrome: Etiologies, pathophysiology, and management. Kidney Int..

[3] Bichon A., Bourenne J., Gainnier M., Carvelli J. (2021). Capillary leak syndrome: State of the art in 2021. Rev. Med. Interne.

[4] Druey K.M. (2010). Narrative Review: The Systemic Capillary Leak Syndrome. Ann. Intern. Med..

[5] Xie Z., Chan E., Yin Y., Ghosh C.C., Wisch L., Nelson C., Young M., Parikh S.M., Druey K.M. (2014). Inflammatory Markers of the Systemic Capillary Leak Syndrome (Clarkson Disease). J. Clin. Cell. Immunol..

[6] Guffroy A., Dervieux B., Gravier S., Martinez C., Deibener-Kaminsky J., Hachulla E., Michel M., Weber J.-C., Korganow A.-S., Arnaud L. (2017). Systemic capillary leak syndrome and autoimmune diseases: A case series. Semin. Arthritis Rheum..

[7] Shin J.I., Lee K.H., Lee I.R., Oh J.H., Kim D.W., Shin J.W., Eo T.S., Kronbichler A., Eisenhut M., Van der Vliet H.J. (2018). Systemic Capillary Leak Syndrome (Clarkson Syndrome) in Cancer Patients: A Systematic Review. J. Clin. Med..

[8] Xie Z., Ghosh C.C., Patel R., Iwaki S., Gaskins D., Nelson C., Jones N., Greipp P.R., Parikh S.M., Druey K.M. (2012). Vascular endothelial hyperpermeability induces the clinical symptoms of Clarkson disease (the systemic capillary leak syndrome). Blood.

[9] Kamal A.H., Gonzalez-paz N.C., Kumar S., Greipp P.R. (2010). Idiopathic Systemic Capillary Leak Syndrome (Clarkson’s Disease): The Mayo Clinic Experience. Mayo Clin. Proc..

[10] Izzedine H., Mathian A., Amoura Z., Ng J.H., Jhaveri K.D. (2022). Anticancer Drug-Induced Capillary Leak Syndrome. Kidney Int. Rep..

[11] Feng J., Shao M., Hu Y., Huang H. (2022). Profile of capillary-leak syndrome in patients received chimeric antigen receptor T cell therapy. Bone Marrow Transplant..

[12] Houterman M., Ellenbroek D., Humalda J.K., van der Hoeven J.G., Ramakers B.P. (2022). Diagnostic and therapeutic considerations in idiopathic systemic capillary leak syndrome: A case report. J. Emerg. Crit. Care Med..

[13] de Chambrun M.P., Luyt C.-E., Beloncle F., Gousseff M., Mauhin W., Argaud L., Ledochowski S., Moreau A.-S., Sonneville R., Verdière B. (2017). The Clinical Picture of Severe Systemic Capillary-Leak Syndrome Episodes Requiring ICU Admission. Crit. Care Med..

[14] Fardet L., Kerob D., Rybojad M., Pennamen V., Schlemmer B., Guermazi A., Morel P., Lebbé C. (2004). Idiopathic Systemic Capillary Leak Syndrome: Cutaneous Involvement Can Be Misleading. Dermatology.

